# Molecular Mechanism of Epimedium Extract against Ischemic Stroke Based on Network Pharmacology and Experimental Validation

**DOI:** 10.1155/2022/3858314

**Published:** 2022-10-27

**Authors:** Hongbei Xu, Mingyao You, Xiang Xiang, Jun Zhao, Ping Yuan, Lan Chu, Chenchen Xie

**Affiliations:** ^1^Department of Neurology, The Affiliated Hospital of Guizhou Medical University, Guizhou 550004, China; ^2^Neurosurgery Department of Chongqing University, Three Gorges Hospital, Chongqing 400010, China; ^3^Department of Neurosurgery, Dazhou Hospital of Integrated Traditional and Western Medicine, 635000, China; ^4^Department of Neurology, Affiliated Hospital & Clinical Medical College of Chengdu University, Chengdu 610081, China; ^5^Department of Neurology, The Second Affiliated Hospital of Chongqing Medical University, Chongqing 400010, China

## Abstract

Ischemic stroke exhibits high morbidity, disability, and mortality, and treatments for ischemic stroke are limited despite intensive research. The potent neuroprotective benefits of Epimedium against ischemic stroke have gained lots of interest. Nevertheless, systematic research on the direct role and mechanisms of Epimedium in ischemic stroke is still lacking. Network pharmacology analysis coupled with experimental verification was utilized to systematically evaluate the potential pharmacological mechanism of Epimedium against ischemic stroke. The TCMSP database was used to mine the bioactive ingredients and Epimedium's targets. The DrugBank, OMIM, and GeneCards databases were employed to identify potential targets of ischemic stroke. GO and KEGG pathway analyses were also carried out. The interaction between active components and hub targets was confirmed via molecular docking. An experimental ischemic stroke model was used to evaluate the possible therapeutic mechanism of Epimedium. As a result, 23 bioactive compounds of Epimedium were selected, and 30 hub targets of Epimedium in its function against ischemic stroke were identified, and molecular docking results demonstrated good binding. The IL-17 signaling pathway was revealed as a potentially significant pathway, with the NF-*κ*B and MAPK/ERK signaling pathways being involved. Furthermore, in vivo experiments demonstrated that Epimedium treatment could improve neurological function and reduce infarct volume. Additionally, Epimedium reduced the activation of microglia and astrocytes in both the ischemic penumbra of the hippocampus and cerebral cortex following ischemic stroke. Western blot and RT–qPCR analyses demonstrated that Epimedium not only depressed the expression of IL-1*β*, TNF-*α*, IL-6, and IL-4 but also inhibited the NF-*κ*B and MAPK/ERK signaling pathways. This study applied network pharmacology and in vivo experiment to explore possible mechanism of Epimedium's role against ischemic stroke, which provides insight into the treatment of ischemic stroke.

## 1. Introduction

Stroke is the second leading cause of death and the third leading cause of disability among adults worldwide; thus, stroke not only is a major public health problem but also confers a huge social and economic burden [1]. Ischemic stroke accounts for the highest proportion of all stroke types, up to 70%–80% [2]. The only FDA-approved medication for the treatment of acute ischemic stroke is a recombinant tissue plasminogen activator (rt-PA). However, the narrow time window and its side effects severely limited its clinical application [3]. Therefore, the development of complementary and alternative therapies is crucial.

For more than 2000 years, traditional Chinese medicine (TCM) has been utilized extensively to treat a variety of diseases, including ischemic stroke. The multiple targets methodology of TCM has been proposed to be helpful in reducing the development of ischemic stroke due to the complicated pathological processes. Epimedium, a member of the Berberidaceae family [4], has a broad range of biological activities and contains a variety of flavonoids [5]. Recent research has demonstrated the effectiveness of formulations containing Epimedium in treating cerebral diseases, including ischemic brain diseases, Alzheimer's dementia, vascular dementia, aging, and depression [6–11]. Our previous study showed that Epimedium exerts a protective effect against vascular dementia [11]. The active ingredients of Epimedium, icariin [12], quercetin [13], kaempferol [14], and luteolin [15], have also been shown in modern pharmacological research to alleviate ischemic stroke injury. Nevertheless, a systematic study on the direct effect and mechanisms of Epimedium in ischemic stroke is currently lacking.

Given the potentially important role of Epimedium in ischemic stroke, it is imperative to explore its mechanism of action further, which may provide more evidence for its clinical application. Network pharmacology, which is based on the systems biology theory fused with computer technology [16], has a broad application value in therapeutic targets, active ingredient discovery, mechanistic research, preclinical efficacy studies, safety assessment, etc. The core research concept of network pharmacology is consistent with the comprehensive view of TCM [17]. Hence, in this study, an integrative approach involving network pharmacology, molecular docking experiments, and experimental validation was used to identify the mechanism of Epimedium for the treatment of ischemic stroke. A visible graphical abstract that demonstrates the anti-ischemic stroke functions and mechanisms of Epimedium is provided (Supplementary 1).

## 2. Materials and Methods

### 2.1. The Screening of Bioactive Compounds of Epimedium

The bioactive compounds of Epimedium were obtained from the Traditional Chinese Medicine Systems Pharmacology Database and Analysis Platform (TCMSP, http://tcmspw.com/). The Chinese name “Yinyanghuo” was used as the keyword for searching. The drug-likeness (DL) and oral bioavailability (OB) of ADME (absorption, distribution, metabolism, and excretion screening method) were employed to predict the bioactive compounds. This study selected the compounds by setting OB ≥ 30% and DL ≥ 0.18 [18]. The 2D molecular structures were also obtained from the TCMSP.

### 2.2. Screening Potential Targets of Epimedium against Ischemic Stroke

The potential targets of Epimedium were acquired from TCMSP (http://tcmspw.com/), and ischemic stroke-related targets were obtained through the DrugBank database (https://www.drugbank.ca/), GeneCards database (https://www.genecards.org/), and OMIM database (https://omim.org/). Subsequently, the potential targets for Epimedium-treated ischemic stroke were obtained from the Venny 2.1.0 platform [19].

### 2.3. Construction of a Protein-Protein Interaction (PPI) Network for Targets

The mapped targets of Epimedium against ischemic stroke were used to construct a PPI network via the STRING database (https://string-db.org/). In that database, the minimum required interaction score for protein interactions was set with the highest confidence score (0.900). The PPI network from STRING was subsequently imported into the Cytoscape 3.8.2 app to investigate the topology parameters, such as ‘betweenness centrality,' ‘closeness centrality,' ‘clustering coefficient,' and ‘degree.' The value of degree analyzed by the Analyse Network tool in Cytoscape 3.8.2 was regarded as a key reference for the selection of hub targets, with the upper limit to maximum degree value and the lowest limit taken as twice the median of degree value [20]. In addition, the molecular complex detection algorithm (MCODE) plugin in Cytoscape was employed to select the highly interconnected cluster with the default parameters.

### 2.4. Gene Ontology (GO) and Kyoto Encyclopedia of Genes and Genomes (KEGG) Pathway Enrichment Analyses

R-language packages ‘ClusterProfiler' and ‘http://org.Hs.eg.Db' were used to analyze the enrichment of GO and KEGG to explore the underlying biological function (BP), cellular components (CC), and molecular function (MF) and signaling pathways. A *p*.adjust (FDR) < 0.05 was considered statistically significant [21]. The bubble chart and Circro circles for GO and KEGG results were visualized by the Sangerbox platform (http://sangerbox.com/Tool). The top ten pathways were selected and used to construct targets-components-pathways network via Cytoscape.

### 2.5. Molecular Docking

The interaction of targets with compounds of Epimedium were predicted by molecular docking. The 2D structures for the compound as a ligand were downloaded from the PubChem database (https://pubchem.ncbi.nlm.nih.gov/), and the 3D structure was generated after minimizing energy in ChemBio 3D software. The receptor protein coded by the core target gene was searched in the UniProt database (https://www.uniprott.org/), and receptor-related entry no. was input into the RCSB PDB database (https://www.rcsb.org/) to download the receptor's 3D molecular structure. PyMOL 2.4.0 software was used to dehydrate the receptor protein, and the AutoGrid tool of AutoDock Tools 1.5.6 was utilized to hydrogenate and generate a docking box. The binding energy of the molecular docking results is a key criterion for the docking effect. The lower the binding energy is, the stronger the molecular docking effect.

### 2.6. Animals

A total of 108 adult male Sprague–Dawley rats (250-300 g) were purchased from the Experimental Animal Center of Chongqing Medical University. All rats were housed under a 12 h light/dark cycle environment with a temperature of 22 ± 2°C and humidity of 65 ± 5%. The experiment complied with the guidelines of the National Institutes for Animal Research and was approved by the Ethics Committee for Animal Experimentation of the Second Affiliated Hospital of Chongqing Medical University.

### 2.7. Construction of the Ischemic Stroke/Reperfusion Model

The model was established according to a previously described method [22]. Briefly, the rats were anaesthetized with 4% isoflurane in 70% N_2_O and 30% O_2_ with a mask [23]. A midline neck incision was made to expose the right external carotid artery (ECA), and a heparin-dampened monofilament nylon suture (Ethicon Nylon Suture; Ethicon Inc., Osaka, Japan) was inserted into the right internal carotid artery (ICA) 2.0 mm from the bifurcation of the right common carotid artery (CCA) to block the right middle cerebral artery (MCA). After 2 h of occlusion, the filament was removed for reperfusion, and ECA was ligated to close the wound. The rats in the sham group underwent all the same surgical procedures except the insertion of sutures. A laser-Doppler flowmeter (PeriFlux 5000, Perimed AB, Sweden) was used to monitor regional cerebral blood flow reduction and restoration. Successful occlusion was confirmed by a decrease in the regional cerebral blood flow to 20% and recovered to more than 80% of the baseline. All rats were closely monitored and maintained on top of a warming pad during the procedures. After recovering from anesthesia, the Longa score [24] was applied to evaluate neurological deficits, and rats with a score of 2 or 3 were included in the subsequent experiment [25].

### 2.8. The Preparation and Treatment of Epimedium

Epimedium extract (LY-0014, Hunan Warrant Pharmaceutial Co., Ltd.) with 98% purity was purchased and diluted into 50 mg/ml concentration with distilled water. All animals were divided into six groups as follows: sham, I/R, I/R+control, I/R+drug (low dose), I/R+drug (medium dose), and I/R+drug (high dose). Rats in the I/R+control group were treated intragastrically with the same volumn of distilled water daily. Rats in the I/R+drug (low dose), I/R+drug (medium dose), and I/R+drug (high dose) groups were administered 50 mg/kg, 100 mg/kg, and 200 mg/kg Epimedium intragastrically daily, respectively [26].

### 2.9. Neurobehavioral Assessment

At 72 hours after reperfusion, the Longa score was used to assess neurobehavioral function [24, 27]. The person who performed these tests was blinded to all rats. The Longa score was graded as follows: 0, no neurological deficits; 1, failure to fully extend the left forepaw; 2, difficulty to extend the left forelimb and circle to the left side; 3, failure to the left side; and 4, no spontaneous walking or decreased level of consciousness [24]. The modified Bederson score was graded as follows: 0, no observable deficits; 1, lost forelimb flexion; 2, lost forelimb flexion with lower resistance to lateral push; 3, unidirectional circling; 4, longitudinal spinning or seizure activity; and 5, no movement [28]. The modified Garcia score was graded from the following six aspects: symmetry of limbs, spontaneous activity, forepaw outstretching, climbing, body proprioception, and response to vibrissal touch [27].

### 2.10. 2,3,5-Triphenyltetrazolium Chloride (TTC) Staining

TTC staining was used to evaluate cerebral infarction volume [29]. Rats were euthanized at 72 h after ischemic reperfusion, and the brains were quickly harvested and frozen at -20°C for 20 minutes. The brains were coronally sliced continuously and incubated with 2% TTC for 10 min at 37°C. The sections were fixed in 10% paraformaldehyde and analyzed with Image-Pro Plus (version 6.0, Media Cybernetics Co., USA). The infarct volume was calculated as follows: percentage hemisphere lesion volume (%HLV = {[total infarct volume − (right hemisphere volume − left hemisphere volume)]/left hemisphere volume} × 100%).

### 2.11. Measurement of Reactive Oxygen Species (ROS)

The brain tissue of the ischemic cortex and hippocampus was extracted rapidly at 72 h following reperfusion. Following the instructions, the ROS assay kit (Cell Biolabs, Inc., USA) was used to quantify the production of intracellular ROS. By using a microplate reader, the observation fluorescence of ROS was measured at 485 nm. The normal control group served as the basis for calculating the ROS content of each experimental group.

### 2.12. Immunofluorescence

Immunofluorescence was performed by a method described previously [29]. After anesthesia, 0.9% saline and 4% formaldehyde were transcardially infused into rats. The brains were carefully removed and dehydrated by 15% and 30% sucrose. Rats were anaesthetized and transcardially perfused with 0.9% saline and 4% formaldehyde. The brains were removed carefully and dehydrated with 15% sucrose and 30% sucrose. Ten-micrometer-thick coronal brain sections were harvested and incubated with 1% Triton X-100 for 30 min at room temperature. Subsequently, the sections were blocked for 1 h at 37°C with 5% bovine serum albumin and then incubated with anti-GFAP mouse antibody (A00213, BOSTER Co.) and anti-Iba-1 goat antibody (NB100-1028, Novus Co., USA, 1 : 200) at 4°C overnight. The next day, the sections were incubated with FITC-conjugated AffiniPure donkey anti-goat IgG (H+L; SA00003-3, Proteintech, 1 : 200) or Alexa Fluor 488-conjugated goat anti-mouse IgG (H+L; SA00006-1, Proteintech, 1 : 200). The DAPI was used to stain nuclei (Sigma, USA, 1 : 200). The images were taken using an A1+R laser confocal microscope (Nikon, Tokyo, Japan).

### 2.13. Western Blot

The brain tissue in the ischemic cortex and hippocampus was extracted rapidly at 72 h after reperfusion. Samples were homogenized in ristocetin-induced platelet aggregation lysis buffer plus PMSF (Beyotime, Shanghai, China), and protein was extracted from the supernatant after centrifugation. Nuclear and cytoplasmic proteins were extracted using a Nuclear and Cytoplasmic Protein Extraction Kit (no. AR0106, Boster, Beijing, China), respectively. The concentration of protein was assayed by a BCA kit (Beyotime, Shanghai, China). Protein samples were loaded and separated by SDS–PAGE and transferred to polyvinylidene fluoride membranes (Millipore Co., USA). The membranes were treated with the respective primary antibodies after blocking in 5% skimmed milk for 2 hours: anti-ERK1/2 rabbit antibody (no. 4695, CST, USA, 1 : 1000), anti-p-ERK1/2 rabbit antibody (no. 4370, CST, USA, 1 : 2000), anti-p38MAPK rabbit antibody (no. 8690, CST, USA, 1 : 1000), anti-p-p38MAPK rabbit antibody (no. ab4822, Abcam, USA, 1 : 1000), anti-NF-*κ*B p65 rabbit antibody (no. 8242, Cell Signaling Technology, USA, 1 : 1000), anti-I*κ*B*α* rabbit antibody (no. 4812, Cell Signaling Technology, USA, 1 : 1000), anti-*β*-actin rabbit antibody (no. 4970, CST, USA, 1 : 1000), and anti-Lamin B rabbit antibody (no. 4970, CST, USA, 1 : 1000) at 4°C overnight. The next day, the membranes were incubated with horseradish peroxidase-conjugated secondary antibody for 1 h at 37°C and scanned by a gel imaging instrument (Vilber Lourmat Fusion FX 7 Spectra, France). The results were analyzed by software (FUSION-CAPT, France). The relative protein content was normalized against *β*-actin or Lamin B.

### 2.14. Real-Time Quantitative Reverse Transcription Polymerase Chain Reaction (RT–qPCR)

Total RNA in the cerebral cortex and hippocampus was isolated using TRIzol (Takara Biotechnology, Japan), and the concentration of each sample was detected by a NanoDrop 2000 spectrophotometer (Thermo Scientific, Bremen, Germany). The mRNA acted as a template to synthesize cDNA by using PrimeScript™ RT reagent kit with gDNA Eraser (TaKaRa) at 42°C for 2 min. Subsequently, the RT–qPCR was analyzed in the iQ5 Gradient Real-Time PCR detection system (Bio-Rad Co., USA). The relative mRNA content was normalized to the housekeeping gene *β*-actin. The sequences of primers for each gene detected are listed in Table 1.

### 2.15. Statistical Analysis

All data are expressed as the mean ± SEM. The Kolmogorov-Smirnov test and Levene's test were used to determine the normality and homogeneity of the variance. Data were assayed using one-way analysis of variance (ANOVA) followed with the Bonferroni post hoc test. Statistical analyses were performed by SPSS 20.0. Statistical significance was set at *p* < 0.05.

## 3. Results

### 3.1. Epimedium Exerted Neuroprotective Effects against Ischemic Stroke in Rats

**Figure 1:**
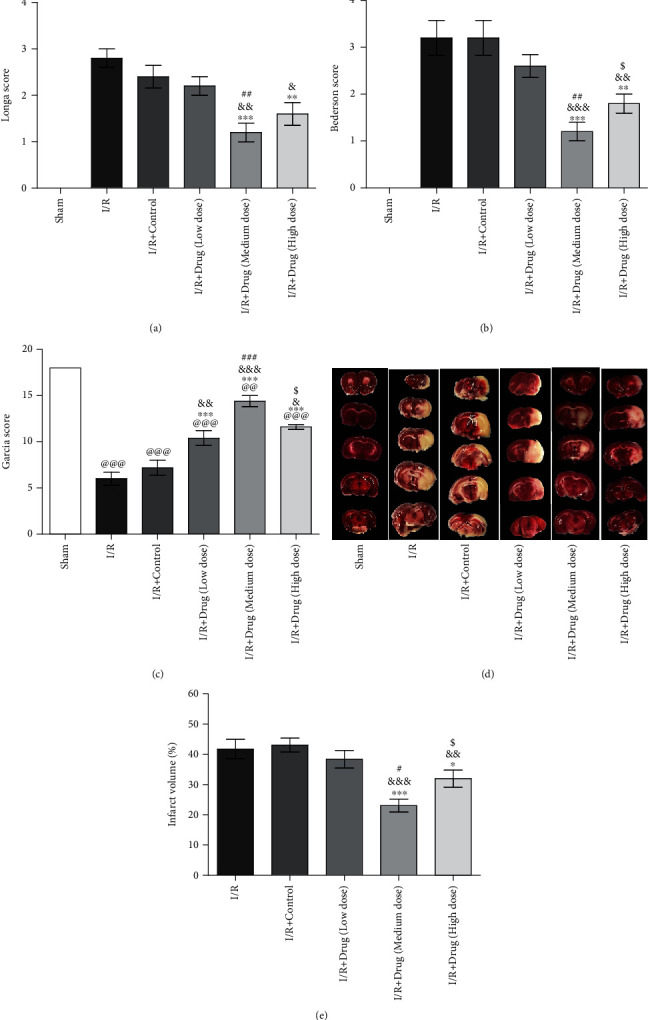
Epimedium exerted a neuroprotective effect in ischemic stroke rats. Neurofunction was evaluated using the Longa score (a, *n* = 5), modified Bederson score (b, *n* = 5), and modified Garcia score (c, *n* = 5). (e, *n* = 5) The quantitative analysis of the cerebral infarct volume detected by TTC (d). ^@@@^*p* < 0.001 and ^@@^*p* < 0.01 versus the sham group; ^∗∗∗^*p* < 0.001, ^∗∗^*p* < 0.01, and ^∗^*p* < 0.05 versus the I/R group; ^&&&^*p* < 0.001, ^&&^*p* < 0.01, and ^&^*p* < 0.05 versus the I/R+control group; ^###^*p* < 0.001, ^##^*p* < 0.01, and ^#^*p* < 0.05 versus the I/R+drug (low dose) group; ^$^*P* < 0.05 versus the I/R+drug (medium dose) group.

Rats were divided into the following 6 groups: the sham, I/R, I/R+control, I/R+drug (low dose), I/R+drug (medium dose), and I/R+drug (high dose) groups. Seventy-two hours after reperfusion, the Longa score, modified Bederson score, and modified Garcia score were used to evaluate neurological deficits. Unlike those in the sham group, rats in the I/R and I/R+control groups exhibited obvious neurological deficits. Rats in the I/R+drug (medium dose) group showed obvious improvements in neurological function compared with rats in the I/R, I/R+control, and I/R+drug (low dose) groups (Figures 1(a)–1(c)). Compared with the I/R+drug (medium dose) group, the I/R+drug (high dose) group had a higher Bederson score and a lower Garcia score (Figures 1(b) and 1(c)). As shown in Figures 1(d) and 1(e), no cerebral infarct was detected in the sham group. The infarct volumes in the I/R+drug (medium dose) and I/R+drug (high dose) groups were decreased compared to those in the I/R, I/R+control, and I/R+drug (low dose) groups. The infarct volume in the I/R+drug (medium dose) group was clearly decreased compared with that in the I/R+drug (high dose) group.

### 3.2. Bioactive Compounds and Targets of Epimedium

A total of 130 chemical ingredients of Epimedium were obtained from the TCMSP database, and 23 bioactive compounds were identified after filtering by setting oral bioavailability (OB) ≥ 30% and drug‐likeness (DL) ≥ 0.18. The specific molecular ID, molecular name, 2D structure, and values of OB and DL for each active compound are shown in Table 2.

### 3.3. Screening and Identification of Therapeutic Targets of Epimedium in Ischemic Stroke

**Figure 2:**
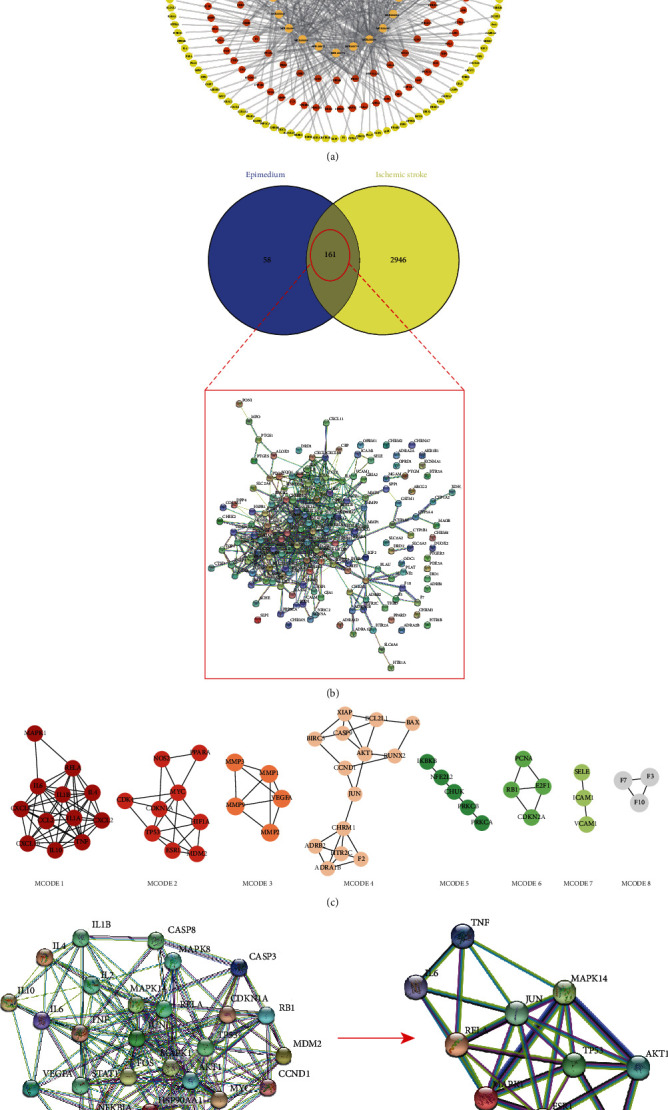
The identification of potential targets of Epimedium for the treatment of ischemic stroke. (a) The bioactive ingredients-targets network of Epimedium. (b) Venn diagram of the overlapping targets of Epimedium and ischemic stroke and the PPI network of the 161 targets. (c) Eight clusters were obtained in the PPI network. (d) The PPI network of hub targets. The hub targets of Epimedium with therapeutic potential for ischemic stroke were identified using Cytoscape software.

A total of 219 gene targets of the pharmacological activity of Epimedium were selected from the TCMSP database. Combined with the 23 bioactive compounds, they were used to establish the ingredient-target network (Figure 2(a)). A total of 3107 therapeutic targets in ischemic stroke were obtained from the DrugBank, OMIM, and GeneCards databases after duplicate targets were deleted. Venn diagram analysis revealed 161 intersecting targets of Epimedium against ischemic stroke, and a protein-protein interaction (PPI) network consisting of the 161 targets was constructed with the STRING database (Figure 2(b)). The targets in the PPI network were further analyzed by MCODE, and 8 clusters were detected. The details are shown in Table 3. The topological parameters of the 161 interacting targets were calculated with Cytoscape, and the minimum and maximum values of degree were 1 and 37, respectively. The screening criteria were set to 14 (twice medium degree) and 37, and a total of 30 hub targets were identified (Figure 2(D)). The PPI network of 30 hub targets of Epimedium with therapeutic potential for ischemic stroke were identified using Cytoscape software (supplementary 2). The PPIs among the top 10 hub targets were further established (Figure 2(D)). The betweenness centrality, closeness centrality, clustering coefficient, and degree of topological parameters for each hub gene are shown in Table 4.

### 3.4. Gene Ontology Enrichment Analysis

**Figure 3:**
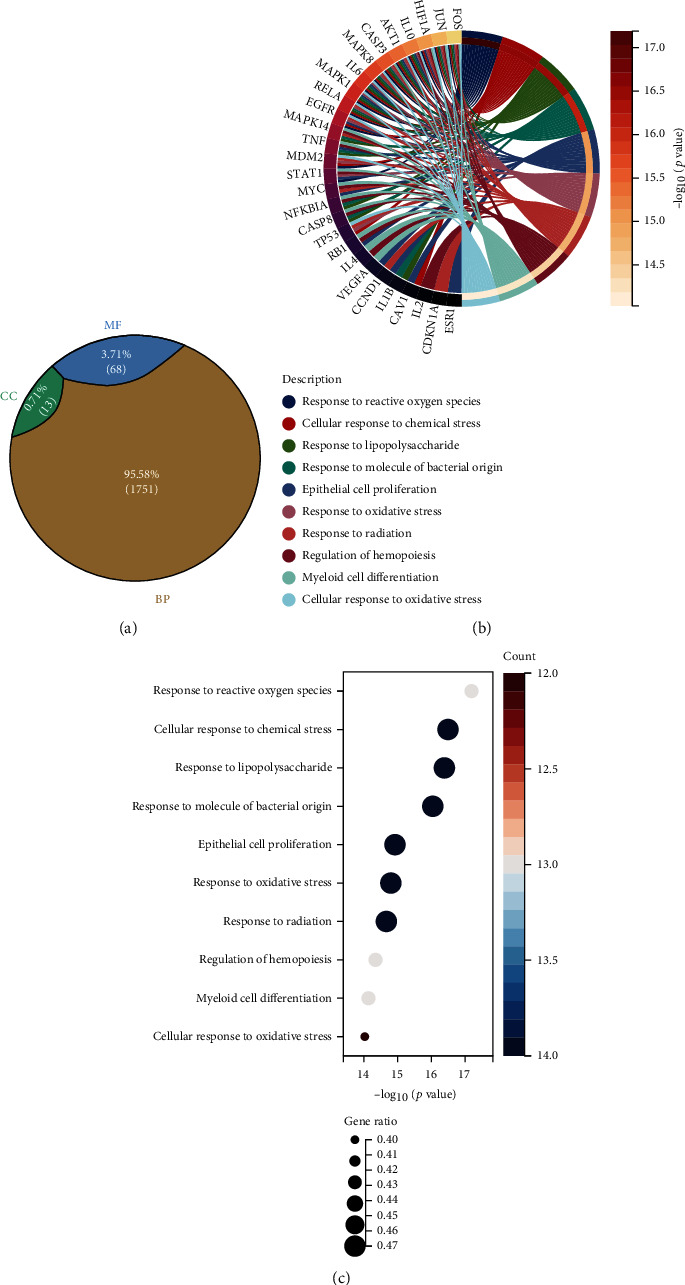
The ratio of GO enrichment analysis and BP associated with hub targets of Epimedium against ischemic stroke. (a) BP has the highest ratio of 95.58%. CC and MF consist of 0.71% (13) and 3.71% (68), respectively. (b) The Circro diagrams of BP. (c) Bubble diagrams of BP was showed with BP term, −log10(*p* value), gene count, and gene ratio.

**Figure 4:**
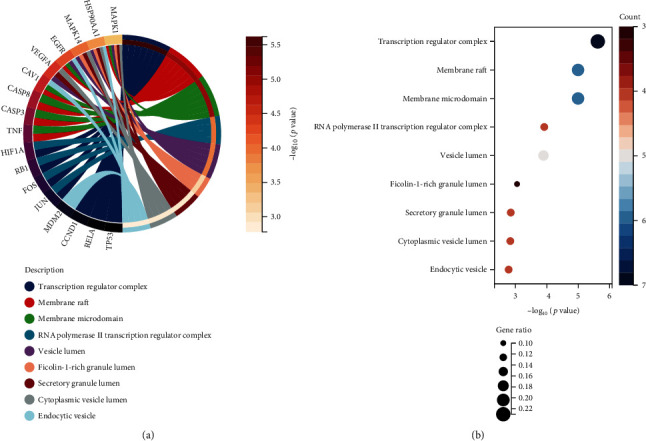
CC associated with hub targets of Epimedium against ischemic stroke. (a) The Circro diagrams of CC. (b) Bubble diagrams of CC was showed with CC term, −log10(*p* value), gene count, and gene ratio.

**Figure 5:**
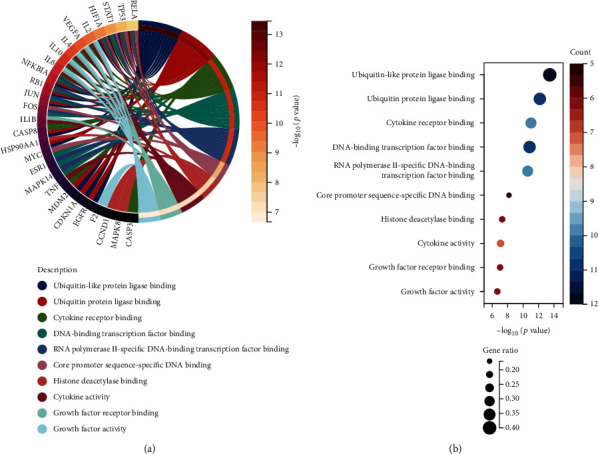
MF associated with hub targets of Epimedium against ischemic stroke. (a) The Circro diagrams of MF. (b) Bubble diagrams of MF was showed with MF terms, −log10(*p* value), gene count, and gene ratio.

The hub Epimedium targets against ischemic stroke were subjected to Gene Ontology (GO) enrichment analysis. A total of 1832 items were produced from the GO enrichment analysis results (Figure 3(a)). The top ten biological process (BP) terms (Figures 3(b) and 3(c)) were the response to reactive oxygen species, the cellular response to chemical stress, the response to lipopolysaccharide, the response to molecules of bacterial origin, epithelial cell proliferation, the response to oxidative stress, the response to radiation, the regulation of haemopoiesis, myeloid cell differentiation, and the cellular response to oxidative stress. The top ten cellular component (CC) terms (Figure 4) were ranked as follows: the transcription regulator complex, membrane rafts, membrane microdomains, the RNA polymerase II transcription regulator complex, the vesicle lumen, the ficolin-1-rich granule lumen, the secretory granule lumen, the cytoplasmic vesicle lumen, endocytic vesicles, and the cyclin-dependent protein kinase holoenzyme complex. The top ten molecular function (MF) terms (Figure 5) were ubiquitin-like protein ligase binding, ubiquitin protein ligase binding, cytokine receptor binding, DNA-binding transcription factor binding, RNA polymerase II-specific DNA-binding transcription factor binding, core promoter sequence-specific DNA binding, histone deacetylase binding, cytokine activity, growth factor receptor binding, and growth factor activity.

### 3.5. KEGG Pathway Enrichment Analysis

**Figure 6:**
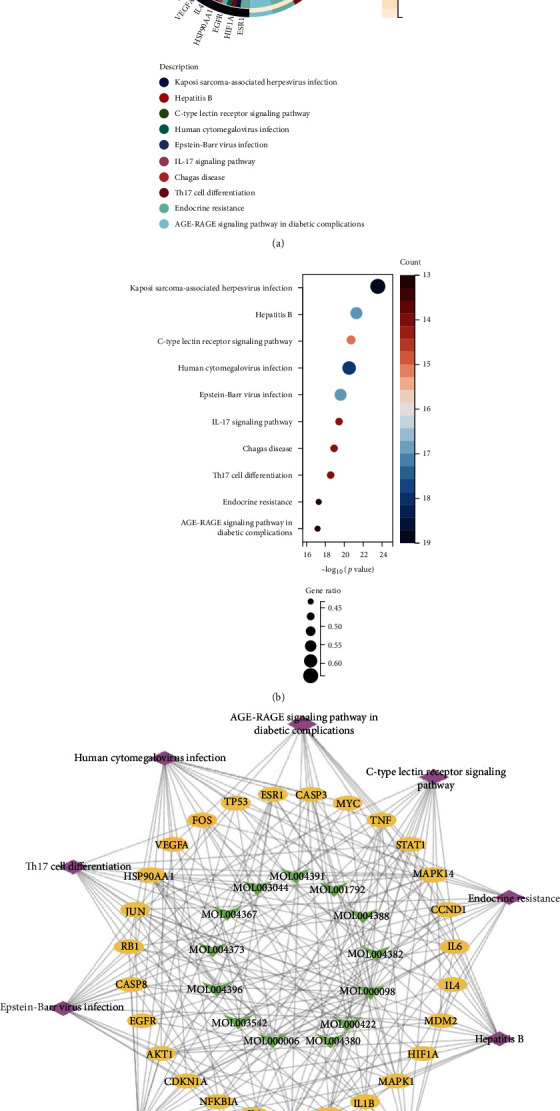
The top ten KEGG pathways of the hub genes were identified by R language. (a) The KEGG pathway is shown by Circro diagrams. (b) The KEGG pathway is shown by bubble diagrams. (c) The network of targets-components-pathways.

The obtained hub targets were used to assay KEGG pathway enrichment. The top ten KEGG signaling pathways (Figure 6) were Kaposi sarcoma-associated herpesvirus infection, hepatitis B, the C-type lectin receptor signaling pathway, human cytomegalovirus infection, Epstein-Barr virus infection, the IL-17 signaling pathway, Chagas disease, Th17 cell differentiation, endocrine resistance, and the AGE-RAGE signaling pathway in diabetic complications. Next, a network consisting of the top ten pathway-related targets and components was constructed, and the following 13 bioactive components were identified: quercetin, luteolin, kaempferol, homoerythrinan, 1,6-didehydro-3,15,16-trimethoxy-(3-beta)-8-isopentenyl-kaempferol, yinyanghuo A, 1,2-bis(4-hydroxy-3-methoxyphenyl)propan-1,3-diol, 8-(3-methylbut-2-enyl)-2-phenyl-chromone, chrysoeriol, DFV, anhydroicaritin, olivil, 6-hydroxy-11,12-dimethoxy-2,2-dimethyl-1, and 8-dioxo-2,3,4,8-tetrahydro-1H-isochromeno[3,4-h]isoquinolin-2-ium (Figure 6(c)).

### 3.6. IL-17 Signaling Pathway

**Figure 7:**
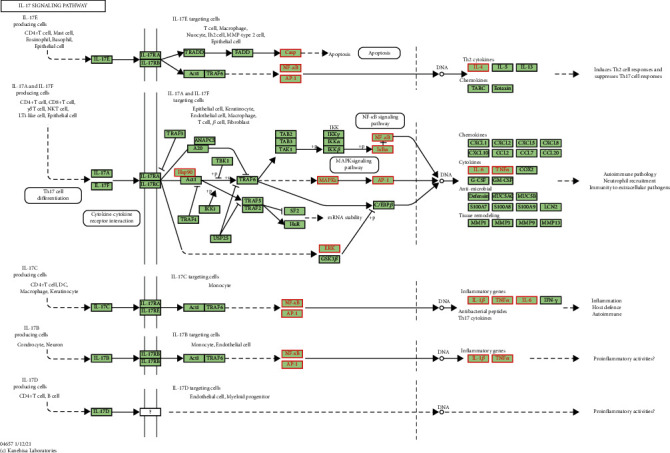
IL-17 signaling pathway. The red targets are the hub genes involved in Epimedium's therapeutic effect on ischemic stroke.

Among the top ten signaling pathways, the IL-17 pathway (Figure 7) plays a potent antineuroinflammatory role induced by ischemic stroke. Among the IL-17 signaling pathway, the NF-*κ*B and MAPK signaling pathways are the key pathways that mediate neuroinflammation.

### 3.7. Results of Molecular Docking Experiments

**Figure 8:**
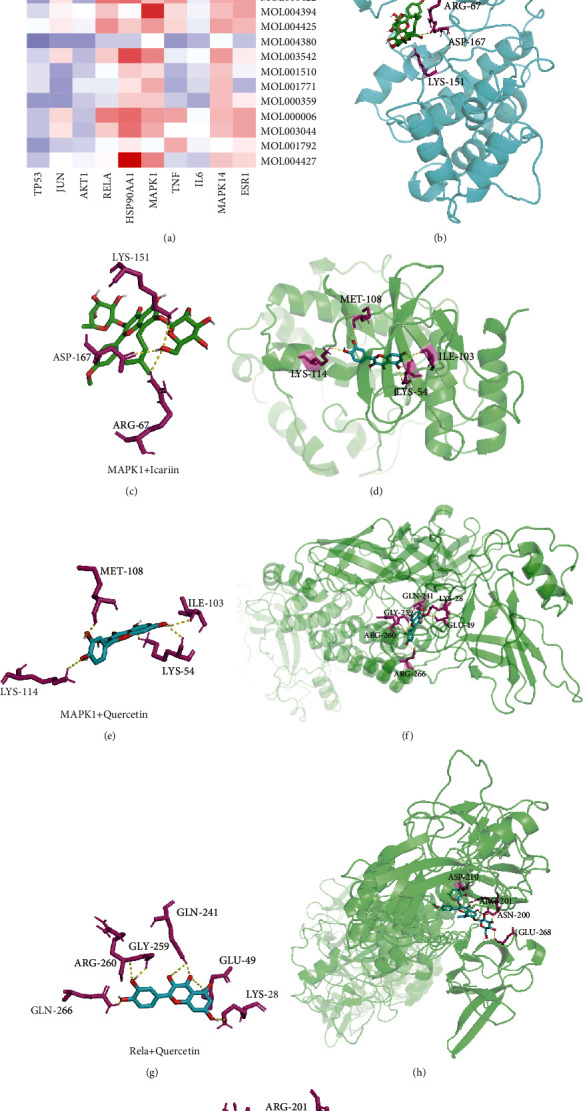
Molecular docking results. (a) The binding energy value in molecular docking. (b, c) MAKP1 docked with icariin. (d, e) MAKP1 docked with quercetin. (f, g) Rela docked with quercetin. (h, i) Rela docked with icariin.

Molecular docking experiments were performed with the top 10 hub targets ranked by degree and 23 molecular components of Epimedium. Binding energies less than -5 kJ·mol^−1^ indicate that the targets bind specifically to the compounds [30]. Molecular docking experiments showed that most of the molecular components of Epimedium have a strong affinity for the top 10 targets (Figure 8(a)). Icariin [31] and quercetin [32] are the two key active ingredients of Epimedium, and molecular docking results suggested that icariin and quercetin bind strongly to MAPK1 and RELA, respectively. Detailed information on the molecular docking experiments is shown in Figure 8(b).

### 3.8. Epimedium Reduced the Oxidative Stress in Rats with Ischemic Stroke

**Figure 9:**
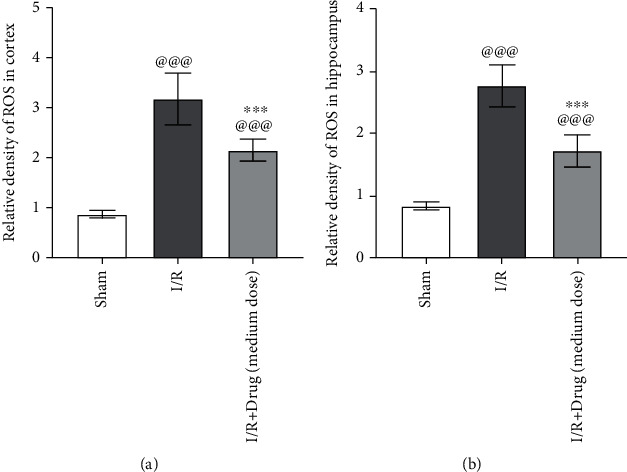
Epimedium reduced the oxidative stress in rats with ischemic stroke. The ROS level in the ischemic cortex (a, *n* = 5) and hippocampus (d, *n* = 5) were analyzed by ROS assay kit. ^@@@^*p* < 0.001 versus the sham group; ^∗∗∗^*p* < 0.001 versus the I/R group.

To investigate the effect of Epimedium on the damage brought about by oxidative stress in cerebral ischemic rats, the levels of ROS were determined. ROS level was significantly higher in both the ischemic cerebral cortex and hippocampus of the I/R group than those in the sham group (Figures 9(a) and 9(b)). After Epimedium treatment, the ROS levels in I/R+drug (medium dose) group was significantly reduced in the ischemic cerebral cortex and hippocampus (Figures 9(a) and 9(b)), indicating a protective effect of Epimedium on oxidative stress–elicited damage.

### 3.9. Epimedium Alleviated Microglial and Astrocyte Activation in Rats with Ischemic Stroke

**Figure 10:**
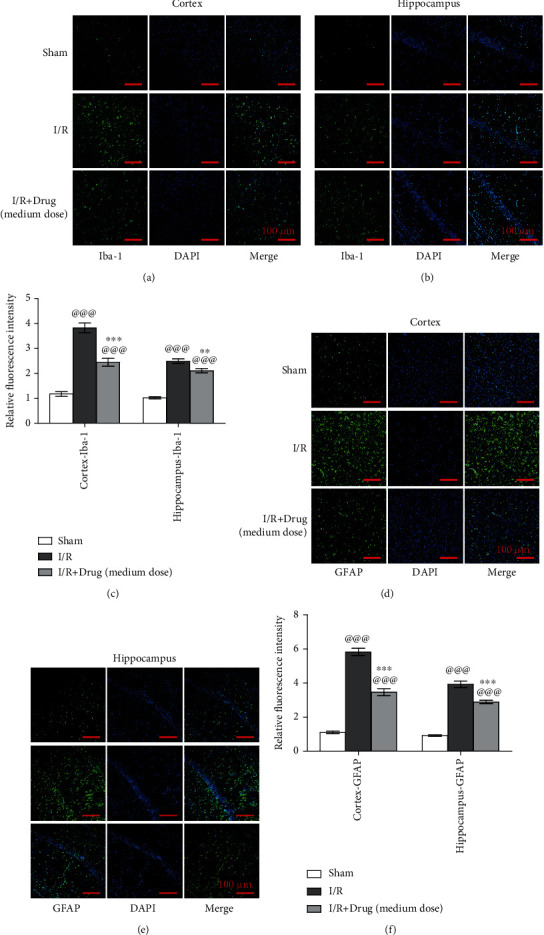
Epimedium alleviated the activation of microglia (Iba-1^+^) and astrocytes (GFAP^+^) in the cortex and hippocampus in ischemic stroke rats. Immunofluorescence was applied to show microglia located in the cortex (a) and hippocampus (b) and astrocytes located in the cortex (d) and hippocampus (e). (c, f) The quantitative analysis of the relative fluorescence intensity of Iba-1 and GFAP (*n* = 6, scale bar = 100 *μ*m). ^@@@^*p* < 0.001 versus the sham group; ^∗∗∗^*p* < 0.001 and ^∗∗^*p* < 0.01 versus the I/R group.

The activation of microglia and astrocytes in the ischemic penumbra of the cortex and hippocampus was detected by immunofluorescence analysis at 72 h after reperfusion. As shown in Figure 10, in the sham group, the microglia located in the cortex and hippocampus were mainly in the resting state, with small cell bodies and branched processes. Microglia in the I/R and I/R+drug (medium dose) groups acquired a reactive phenotype. In both the cortex and hippocampus, the relative fluorescence intensity were significantly greater in the I/R and I/R+drug (medium dose) groups than in the sham group. Compared with that in the I/R group, the relative fluorescence intensity in the I/R+drug (medium dose) group was obviously decreased (Figures 10(a)–10(c)). In the I/R group, the relative fluorescence intensity of GFAP was significantly higher than that in the sham group. After Epimedium treatment, the relative fluorescence intensity of GFAP in the I/R+drug (medium dose) group was significantly lower than that in the I/R group (Figures 10(d) and 10(e)).

### 3.10. Epimedium Modulated the Release of Inflammatory Cytokines and Activation of the MAPK/ERK and NF-*κ*B Signaling Pathways in Rats with Ischemic Stroke

**Figure 11:**
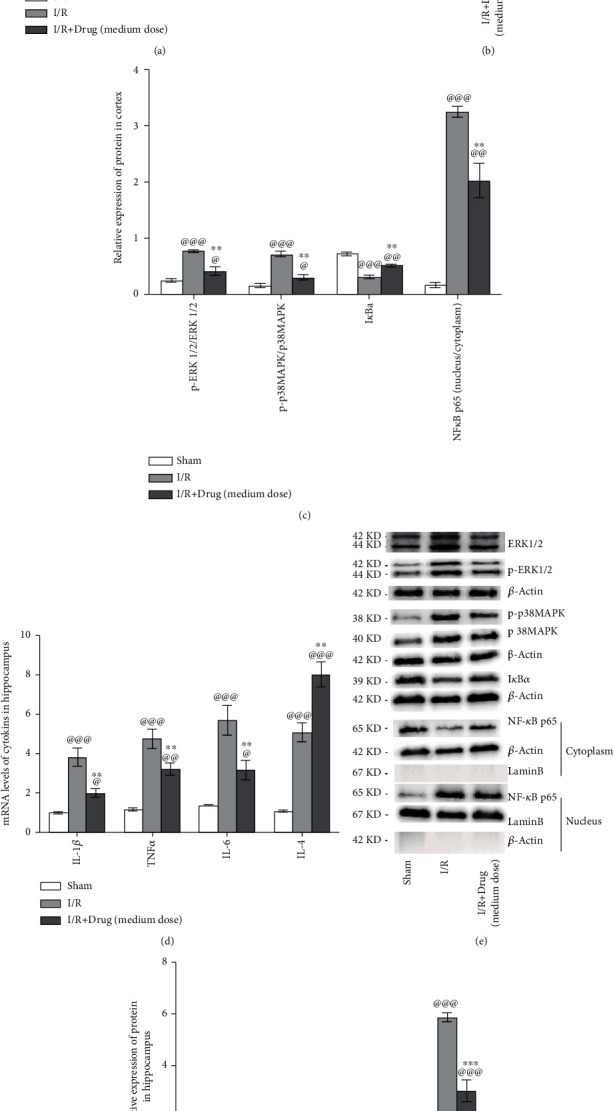
The modulation of inflammatory cytokines and activation of the MAPK/ERK and NF-*κ*B pathways by Epimedium treatment in ischemic stroke rats. RT–qPCR results of TNF-*α*, IL-1*β*, IL-6, and IL-4 levels in the ischemic cortex (a, *n* = 5) and hippocampus (d, *n* = 5). Western blotting results of p-ERK1/2, ERK1/2, p-p38MAPK, p38MAPK, I*κ*B*α*, cytoplasm-p65, and nucleus-p65 proteins expression in the cortex (b, c; *n* = 3) and hippocampus (e, f; *n* = 3). *β*-Actin acted as a total and cytoplasmic protein, while Lamin B acted as a nuclear protein. ^@@@^*p* < 0.001, ^@@^*p* < 0.01, and ^@^*p* < 0.05 versus the sham group; ^∗∗∗^*p* < 0.001, ^∗∗^*p* < 0.01, and ^∗^*p* < 0.05 versus the I/R group.

To investigate the effects of Epimedium treatment on the secretion of pro- and anti-inflammatory mediators, rats were randomly divided into the following three groups: the sham, I/R, and I/R+Epimedium (medium dose) groups. Seventy-two hours after reperfusion, the levels of TNF-*α*, IL-1*β*, IL-6, and IL-4 in the ischemic cerebral cortex and hippocampus were detected by RT–qPCR. The mRNA levels of TNF-*α*, IL-1*β*, and IL-6 in both the ischemic cortex and hippocampus were significantly increased in the I/R group compared with the sham group but obviously decreased in the I/R+Epimedium (medium dose) group. In addition, the mRNA levels of IL-4 were obviously increased in the I/R group compared with the sham group and further increased in the I/R+Epimedium (medium dose) group (Figures 11(a) and 11(d)).

Next, at 72 hours following reperfusion, the MAPK/extracellular signal-regulated kinase (ERK) and NF-*κ*B pathways in the ischemic cerebral cortex and hippocampus were analyzed in order to explore possible mechanisms of Epimedium-mediated neuroprotection after ischemic stroke. Western blotting was used to detect the protein expression of p-ERK1/2, ERK1/2, p-p38MAPK, p38MAPK, I*κ*B*α*, cytoplasmic p65, and nuclear p65. The ratios p-ERK1/2/ERK1/2 and p-p38MAPK/p38MAPK were used to indicate the phosphorylation of ERK1/2 and p38MAPK, respectively. The nuclear/cytoplasmic NF-*κ*B p65 ratio was utilized to represent nuclear translocation of the NF-*κ*B p65 protein. As shown in Figure 11, increased ratios of phosphorylated ERK1/2 and p38MAPK, a decreased I*κ*B*α* content, and increased nuclear translocation of the p65 protein were detected in the I/R group compared to the sham group in both the ischemic cortex (Figures 11(b) and 11(c)) and hippocampus (Figures 11(e) and 11(f)). Compared with those in the I/R group, decreased phosphorylated ERK1/2 and p38MAPK ratios, an increased I*κ*B*α* content, and decreased nuclear translocation of the p65 protein were detected in the I/R+Epimedium (medium dose) group.

## 4. Discussion

Epimedium is an herbal medicine that has been widely used in Korea, Japan, and China. Increasing number of evidence from various ischemic stroke models and species has demonstrated that the active components of Epimedium, such as icariin [12], quercetin [13], kaempferol [14], and luteolin [15], exhibit positive effects on ischemic stroke based on their antioxidant, antineuroinflammatory, and antiapoptotic effects and reduction of blood-brain barrier (BBB) damage [13–15, 26, 33]. But its direct effect and probable mechanism in the treatment of ischemic stroke are currently unclear. The utilization of multiple components that target different factors to achieve a therapeutic effect is a key characteristic of TCM [34]. As a result, direct administration of Epimedium may prevent ischemic stroke injury via a variety of targets and pathways. The underlying effect and mechanism were thus identified by network pharmacology and experimental validation.

In this study, we established a focal cerebral ischemia/reperfusion model and applied Epimedium at various doses as treatment. The clear neuroprotective effect of Epimedium at 100 mg/kg was detected based on the decreased infarct volume and improved neurological function. Based on the complex components of Epimedium, network pharmacology analysis was used to discover the possible mechanism. By searching the TCMSP database, a total of 23 active components of Epimedium, such as icariin, quercetin [13], kaempferol, and luteolin, which have been demonstrated to be beneficial in ischemic stroke, were selected. Following the filtering of common targets between Epimedium and ischemic stroke, 161 targets were filtered out and utilized to establish a PPI network, and 30 hub targets were ultimately identified. Notably, molecular docking analyses demonstrated that the majority of the bioactive components of Epimedium have a high affinity with the top ten hub targets.

Meanwhile, KEGG pathway analysis revealed that the top ten KEGG signaling pathways included classic pathways, such as the C-type lectin receptor signaling pathway, human cytomegalovirus infection, the IL-17 signaling pathway, Th17 cell differentiation, and endocrine resistance. Importantly, by establishing the top ten pathways related to the target-component-pathway network, 13 bioactive components were further selected. Among the 13 components, quercetin, luteolin, and kaempferol have been well demonstrated to exert important neuroprotective effects. Quercetin alleviates ischemic brain injury through its antioxidant, antiapoptotic, and antineuroinflammation activities and ability to reduce BBB damage [13] and regulates the phosphorylation of ERK [35]. Tan et al. [15] demonstrated that luteolin could reduce the activation of glial cells in the hippocampus after ischemic stroke. Kaempferol was reported to reduce glial activation-mediated inflammation, alleviate neuronal injury by enhancing autophagy, and decrease BBB damage in rats with ischemic stroke by regulating the NF-*κ*B pathway [14, 36]. Therefore, to some extent, our study demonstrates that the active components of Epimedium form the material basis of the mechanism of Epimedium in ischemic stroke.

Ischemic stroke is mainly caused by a reduction in blood flow to the brain parenchyma which has complex pathophysiological mechanisms [37]. This intricate process includes bioenergy failure, excitotoxicity, oxidative stress, inflammation, the immune reaction, apoptosis, and imbalance of ion homeostasis [38–42]; however, the intricate, multifactorial mechanism is currently not well understood. In this study, GO enrichment analysis showed a classic response to ROS, a cellular response to chemical stress, a response to lipopolysaccharide, and a response to oxidative stress.

Because of its high metabolic activity and high susceptibility to ischemic damage, the brain is particularly vulnerable to oxidative damage [39]. Following ischemic stroke, oxidative stress is characterized as a state of imbalance between the generation and removal of free radicals [43, 44]. It also plays a significant role in the pathophysiology of both acute and chronic phases of ischemic stroke [45]. ROS are generated mainly by microglia and astrocytes following ischemic stroke [46]. ROS imbalance has detrimental effects, including apoptosis, autophagy, inflammation, BBB dysfunction, and cerebral edema [45]. Tan et al. [15] proved that luteolin treatment could reduce oxidative stress, the inflammatory response, and neuronal apoptosis induced by ischemic stroke, which may be related to regulation of the NF-*κ*B and MAPK pathways. It has been well demonstrated that quercetin, an active component of Epimedium, can significantly reduce infarct size, associated with decreases in the levels of the prooxidative mediator malondialdehyde (MDA) and myeloperoxidase (MPO) and significant increases in the levels of the antioxidative mediators superoxide dismutase (SOD) and catalase (CAT). The current study suggests that Epimedium could directly alleviate oxidative stress injury and reduce ROS production, but further exploration is warranted.

Neuroinflammation plays a predominant role in the complicated pathologies that exacerbate cerebral ischemia/reperfusion injury [47]. Neuroinflammation mainly involves the robust activation of glial cells, the infiltration of peripheral immune cells, and the subsequent generation of inflammatory mediators [48, 49]. The mitigation of overactivated neuroinflammation is essential to improve the prognosis of ischemic stroke. Interestingly, in this study, KEGG results showed that the IL-17 signaling pathway, which plays an inflammation-related role in ischemic stroke, may be a critical mechanism involved in the treatment effects of Epimedium in ischemic stroke. The IL-17 family consists of several cytokines that participate in both acute and chronic inflammatory responses [50]. IL-17 is expressed by both Th17 and *γδ* T cells and is detrimental in stroke [51, 52]. In addition to recruited immune cells of the immune system, CNS-resident astrocytes and microglia are sources of IL-17 production during damage progression in the ischemic brain [50]. Microglia and astrocytes are activated within hours following ischemic stroke, which further triggers the generation of cytokines and chemokines [53–55]. These cells also undergo both morphological and phenotypical changes [56]. Although microglia and astrocytes play dual roles following ischemic stroke, overactivated microglia and astrocytes are always detrimental, which cause uncontrolled inflammation to exacerbate tissue damage and neuronal death [56–58]. The current study showed a rapid activation of microglia and astrocytes following ischemic stroke. Importantly, Epimedium suppressed the activation of microglia and astrocytes in both the ischemic penumbra of the cerebral cortex and the hippocampus. This study reveals that Epimedium can reduce the activation of microglia and astrocytes, but whether this action affects cerebral functions deserve further exploration in future research.

By binding IL-17R, IL-17 mediates inflammatory responses via the NF-*κ*B and MAPK pathways [50, 59]. NF-*κ*B [60, 61] and MAPK/ERK [62] are obviously activated following ischemic stroke and are the main pathways of inflammatory response. Ischemic stroke induces nuclear translocation of the NF-*κ*B p65 protein and the subsequent transcription of proinflammatory cytokine genes [63, 64]. The appropriate inhibition of nuclear translocation of NF-*κ*B p65 protein can effectively inhibit glial cell-induced neuroinflammation following ischemic stroke [65]. Consistent with previous studies [63, 64], the current study reveals significant activation of nuclear translocation of the NF-*κ*B p65 protein following ischemic stroke. Importantly, the results showed that Epimedium administration could directly depress ischemic stroke-induced NF-*κ*B activation, as demonstrated by increased I*κ*B*α* and decreased nuclear translocation of NF-*κ*B p65. Interestingly, icariin [12], luteolin [15], and kaempferol [14], the bioactive components of Epimedium, can also significantly improve neurological injury by inhibiting the DNA-binding activity of NF-*κ*B. On the other hand, the MAPK/ERK signaling pathway is also involved in inflammatory processes and is the main signaling pathway that regulates neuroinflammation following cerebral I/R injury [66, 67]. The inhibition of MAPK cascade via suppression of cytokines through anti-inflammatory drugs, blocking p38 MAPK, arrested the production of TNF-*α* and IL-1*β*, resulting in neuroprotection [67, 68]. Wang et al. found that quercetin could reduce cerebral ischemic injury by suppressing the phosphorylation of ERK [35]. The reduced oxidative stress, inflammatory response, and neuronal apoptosis after ischemic stroke by luteolin treatment may be related to regulation of the MAPK signaling pathway [15]. Importantly, this study showed that the activation of the MAPK/ERK pathway was inhibited by Epimedium treatment. Therefore, we have reason to believe that Epimedium reduces the production and release of proinflammatory factors by inhibiting the MAPK/ERK signaling pathway. In a follow-up study, an antagonist or genetic intervention would be utilized to interfere with the NF-*κ*B and MAPK signaling pathways to further clarify the direct role of NF-*κ*B and MAPK in the ability of Epimedium to alleviate brain injury in ischemic stroke.

Moreover, the KEGG Mapper results further showed that IL-1*β*, TNF-*α*, IL-6, and IL-4 are core potential targets involved in Epimedium-mediated neuroinflammation following ischemic stroke. IL-1*β*, TNF-*α*, and IL-6 are major proinflammatory cytokines [69, 70], while IL-4 acts as an anti-inflammatory cytokine [71]. Consistent with the network pharmacology findings, in the current study, it is revealed that IL-1*β*, TNF-*α*, IL-6, and IL-4 were significantly increased in the cerebral cortex and hippocampus after ischemic stroke. Epimedium treatment significantly inhibited the expression of IL-1*β*, TNF-*α*, and IL-6 and enhanced the expression of IL-4, which suggests that Epimedium exerted its neuroprotective effects via regulating the expression of proinflammatory and anti-inflammatory cytokine following ischemic stroke.

## 5. Conclusion

In summary, our study suggests that Epimedium exerts a neuroprotective effect, oxidative stress, and neuroinflammation, possibly by regulating the MAPK/ERK and NF-*κ*B signaling pathways. Although further detailed pharmacological mechanisms are still required to investigate the direct role of NF-*κ*B and MAPK in the neuroprotective role of Epimedium following ischemic stroke in depth, this study first systematically reveals the mechanism of Epimedium in ischemic stroke through a network pharmacology approach. These results provide further theoretical basis for application of Epimedium in the treatment of ischemic stroke.

## Figures and Tables

**Table 1:** List of primers in RT–qPCR.

Gene	Sequence	
IL-1*β*	Forward	5′-GGGATGATGACGACCTGC-3′
	Reverse	5′-CCACTTGTTGGCTTATGTT-3′
TNF-*α*	Forward	5′-GCCACCACGCTCTTCTGC-3′
	Reverse	5′-GCTACGGGCTTGTCACTCG-3′
IL-6	Forward	5′-CCTGCAGCTGGAGAGTGTGGAT-3′
	Reverse	5′-TGTGCTCTGCTTGTGAGGTGCT-3′
IL-4	Forward	5′-CGTGATGTACCTCCGTGCTT-3′
	Reverse	5′-GGACTGCAAGTATTTCCCTCGT-3′
*β*-Actin	Forward	5′-TGTCACCAACTGGGACGATA-3′
	Reverse	5′- GGGGTGTTGAAGGTCTCAAA-3′

**Table 2:** Basic information of bioactive components of Epimedium.

	Molecular ID	Molecule name	2D structure	OB (%)	DL
1	MOL000622	Magnograndiolide	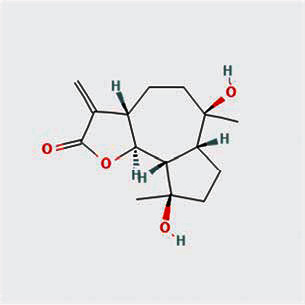	63.71	0.19
2	MOL004367	Olivil	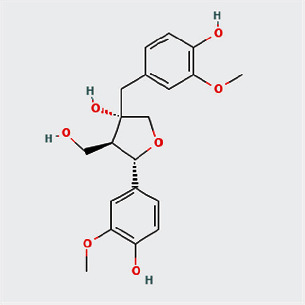	62.23	0.41
3	MOL004388	6-Hydroxy-11,12-dimethoxy-2,2-dimethyl-1,8-dioxo-2,3,4,8-tetrahydro-1H-isochromeno[3,4-h]isoquinolin-2-ium	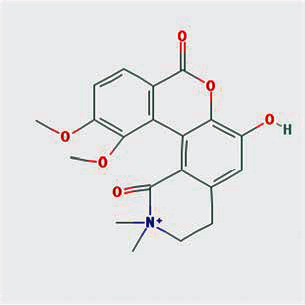	60.64	0.66
4	MOL004382	Yinyanghuo A	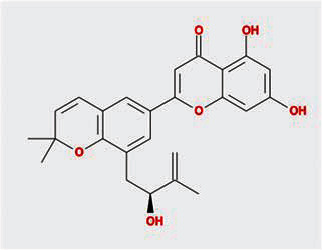	56.96	0.77
5	MOL004396	1,2-Bis(4-hydroxy-3-methoxyphenyl)propan-1,3-diol	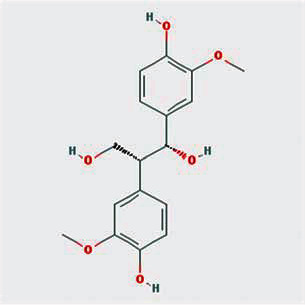	52.31	0.22
6	MOL004386	Yinyanghuo E	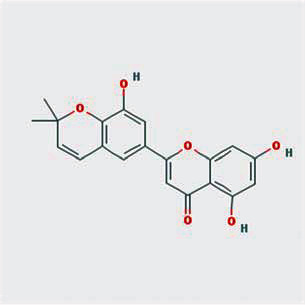	51.63	0.55
7	MOL004391	8-(3-Methylbut-2-enyl)-2-phenyl-chromone	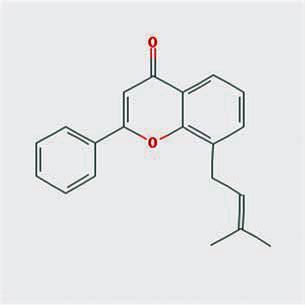	48.54	0.25
8	MOL000098	Quercetin	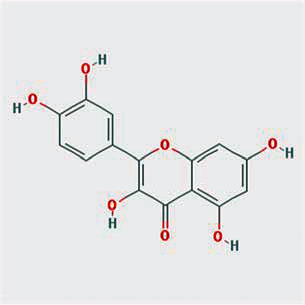	46.43	0.28
9	MOL004384	Yinyanghuo C	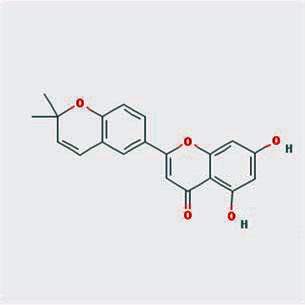	45.67	0.50
10	MOL004373	Anhydroicaritin	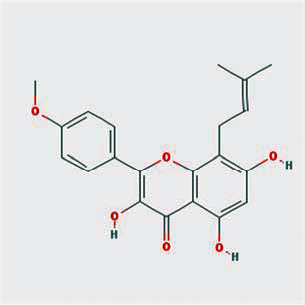	45.41	0.44
11	MOL001645	Linoleyl acetate	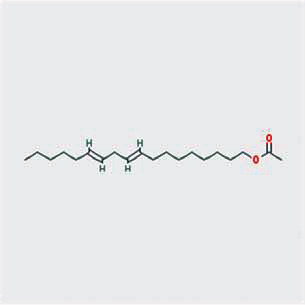	42.10	0.20
12	MOL000422	Kaempferol	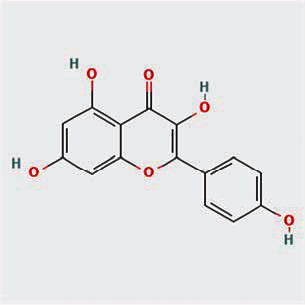	41.88	0.24
13	MOL004394	Anhydroicaritin-3-O-alpha-L-rhamnoside	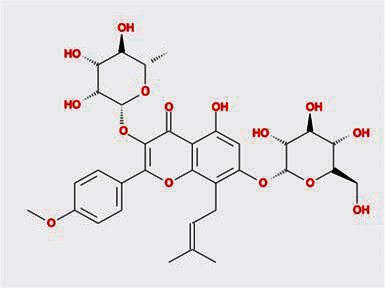	41.58	0.61
14	MOL004425	Icariin	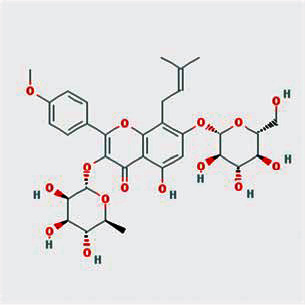	41.58	0.61
15	MOL004380	C-Homoerythrinan, 1,6-didehydro-3,15,16-trimethoxy-, (3.beta.)-	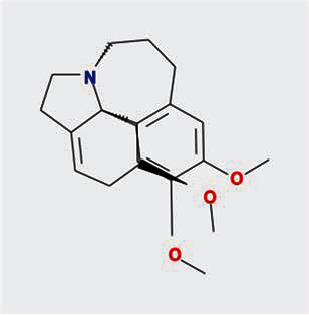	39.14	0.49
16	MOL003542	8-Isopentenyl-kaempferol	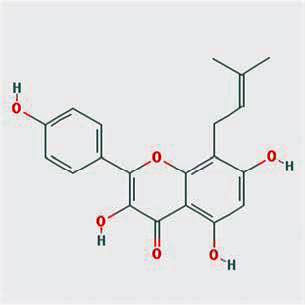	38.04	0.39
17	MOL001510	24-Epicampesterol	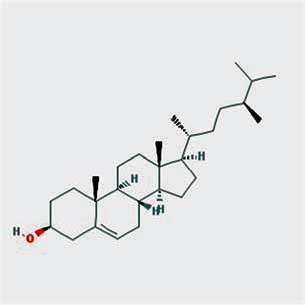	37.58	0.71
18	MOL001771	Poriferast-5-en-3beta-ol	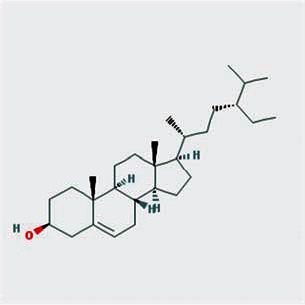	36.91	0.75
19	MOL000359	Sitosterol	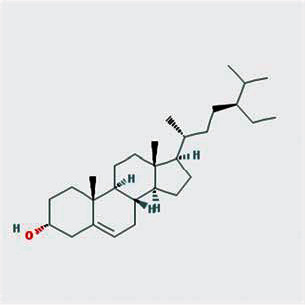	36.91	0.75
20	MOL000006	Luteolin	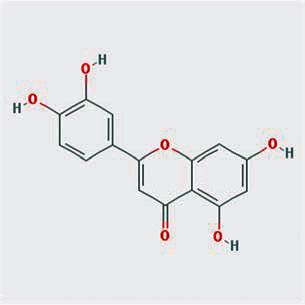	36.16	0.25
21	MOL003044	Chryseriol	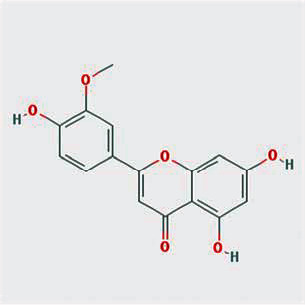	35.85	0.27
22	MOL001792	DFV	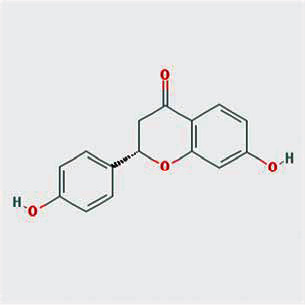	32.76	0.18
23	MOL004427	Icariside A7	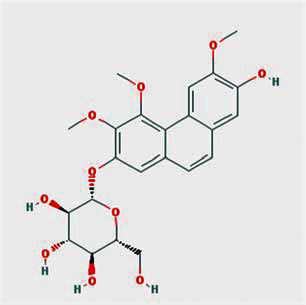	31.91	0.86

**Table 3:** Clusters information of 161 overlapping targets.

MCODE	Score	Nodes	Edges	Gene symbol
1	9.455	12	52	RELA, CXCL2, CCL2, TNF, MAPK1, IL4, CXCL8, CXCL10, IL1A, IL10, IL6, IL1B
2	5	9	20	CDKN1A, HIF1A, MDM2, PPARA, TP53, CDK4, NOS2, ESR1, MYC
3	4.5	5	9	VEGFA, MMP2, MMP3, MMP9, MMP1
4	4	14	26	F2, BCL2L1, CASP9, CHRM1, ADRB2, HTR2C, AKT1, RUNX2, ADRA1B, BAX, JUN, BIRC5, CCND1, XIAP
5	3.5	5	7	PRKCA, CHUK, NFE2L2, PRKCB, IKBKB
6	3.333	4	5	E2F1, CDKN2A, PCNA, RB1
7	3	3	3	ICAM1, VCAM1, SELE
8	3	3	3	F7, F3, F10

**Table 4:** Topological parameters of the hub genes.

Target	Betweenness centrality	Closeness centrality	Clustering coefficient	Degree
TP53	0.11	0.46	0.21	37
JUN	0.12	0.49	0.26	36
AKT1	0.07	0.44	0.19	34
RELA	0.06	0.47	0.28	33
HSP90AA1	0.07	0.46	0.19	32
MAPK1	0.15	0.49	0.23	32
TNF	0.06	0.44	0.30	28
IL6	0.04	0.42	0.34	25
MAPK14	0.02	0.44	0.30	24
ESR1	0.03	0.42	0.32	23
MYC	0.03	0.44	0.42	22
FOS	0.02	0.43	0.32	22
CAV1	0.04	0.43	0.15	19
EGFR	0.03	0.42	0.23	19
RB1	0.02	0.42	0.43	18
CDKN1A	0.01	0.41	0.46	18
MAPK8	0.02	0.41	0.20	18
IL10	0.02	0.36	0.39	18
HIF1A	0.01	0.42	0.50	17
CCND1	0.01	0.39	0.48	17
STAT1	0.02	0.42	0.37	16
CASP3	0.01	0.40	0.36	15
IL4	0.04	0.39	0.54	15
IL1B	0.01	0.39	0.59	15
VEGFA	0.02	0.39	0.30	15
F2	0.15	0.38	0.12	14
IL2	0.01	0.42	0.49	14
MDM2	0.00	0.37	0.42	14
CASP8	0.03	0.40	0.24	14
NFKBIA	0.01	0.42	0.49	14

## Data Availability

The data used to support the findings of this study are available from the corresponding author upon request.

## Abbreviations

ADME: Absorption, distribution, metabolism, and excretion
AGE-RAGE: Advanced glycation end products-receptor for advanced glycation end products
ANOVA: One-way analysis of variance
BBB: Blood-brain barrier
BP: Biological function
CAT: Catalase
CC: Cellular components
DL: Drug-likeness
ECA: External carotid artery
ERK: Extracellular signal-regulated kinase
GO: Gene Ontology
ICA: Internal carotid artery
IL-1*β*: Interleukin-1*β*
IL-4: Interleukin-4
IL-10: Interleukin-10
IL-17: Interleukin-17
I/R: Cerebral ischemia/reperfusion
I*κ*B*α*: Inhibitor of NF-*κ*B*α*
KEGG: Kyoto Encyclopedia of Genes and Genomes
MAPK: Mitogen-activated protein kinase
MCODE: Molecular complex detection algorithm
MCA: Middle cerebral artery
MDA: Malondialdehyde
MF: Molecular function
MPO: Myeloperoxidase
NF-*κ*B: Nuclear factor kappa-B
OB: Oral bioavailability
PPI: Protein-protein interaction
rt-PA: Recombinant tissue plasminogen activator
ROS: Reactive oxygen species
RT–qPCR: Real-time quantitative reverse transcription polymerase chain reaction
SOD: Superoxide dismutase
TCM: Traditional Chinese medicine
TCMSP: Traditional Chinese Medicine Systems Pharmacology
TNF-*α*: Tumor necrosis factor-*α*
TTC: Triphenyltetrazolium chloride.

## Supplementary Materials

Supplementary Materials A visible graphical abstract that demonstrates the anti-ischemic stroke functions and mechanisms of Epimedium is provided.
