# Supplementary material for: Molecular Mechanism of Epimedium Extract against Ischemic Stroke Based on Network Pharmacology and Experimental Validation
Source: Oxid Med Cell Longev. 2022 Oct 27;2022:3858314. doi: 10.1155/2022/3858314 (PMC9633197; doi:10.1155/2022/3858314)
Supplement: Supplementary Materials — A visible graphical abstract that demonstrates the anti-ischemic stroke functions and mechanisms of Epimedium is provided. [file 3858314.f1.zip › supplemental figure1 The flowchart of this study. (1).pdf]

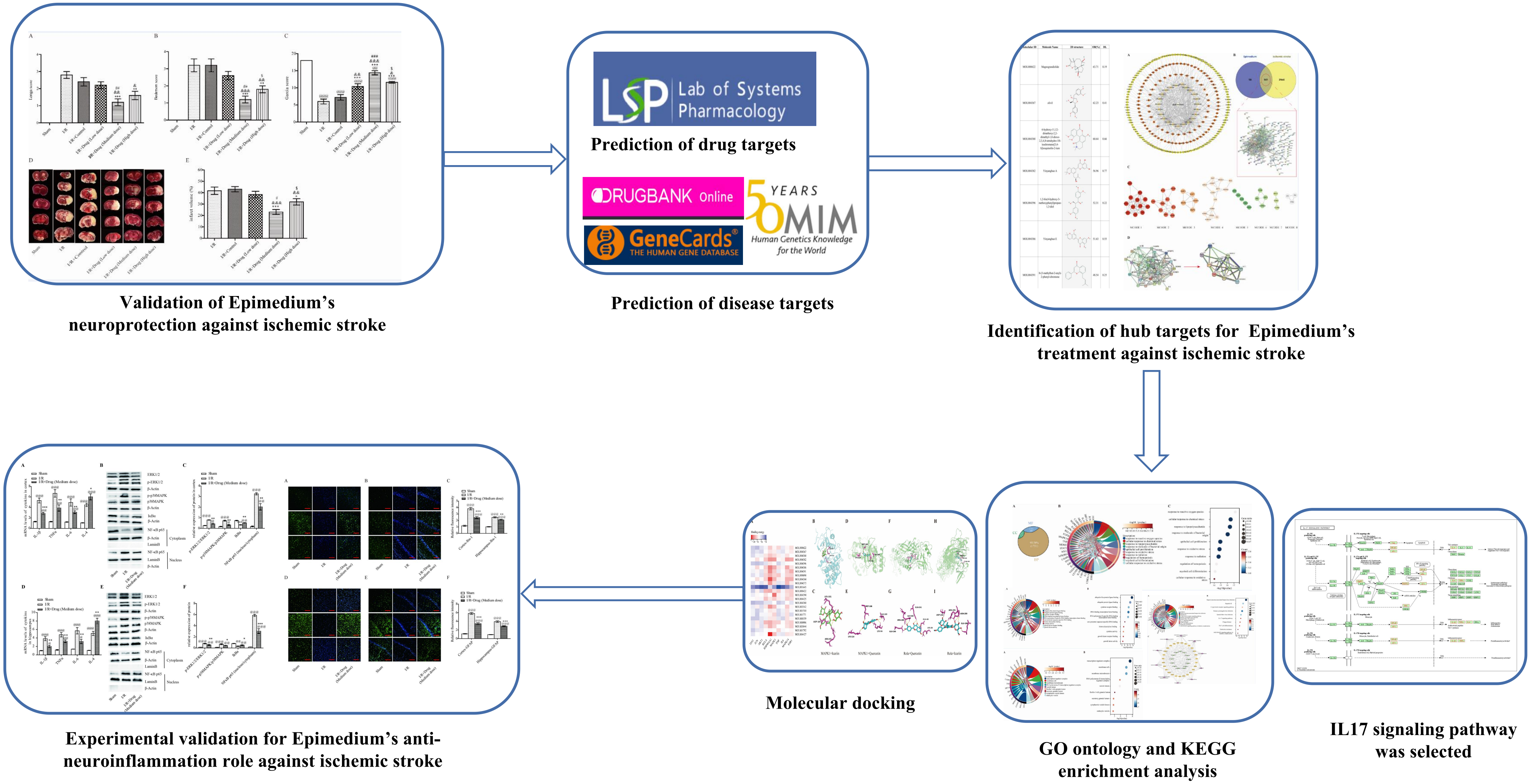
Validation of Epimedium's neuroprotection against ischemic stroke
Lab of Systems Pharmacology
Prediction of drug targets
DRUGBANK Online
GeneCards
THE HUMAN GENE DATABASE
50 YEARS OMIM
Human Genetics Knowledge for the World
Prediction of disease targets
Identification of hub targets for Epimedium's treatment against ischemic stroke
GO ontology and KEGG enrichment analysis
IL17 signaling pathway was selected
Molecular docking
Experimental validation for Epimedium's anti-neuroinflammation role against ischemic stroke
