# Supplementary material for: Molecular Mechanism of Epimedium Extract against Ischemic Stroke Based on Network Pharmacology and Experimental Validation
Source: Oxid Med Cell Longev. 2022 Oct 27;2022:3858314. doi: 10.1155/2022/3858314 (PMC9633197; doi:10.1155/2022/3858314)
Supplement: Supplementary Materials — A visible graphical abstract that demonstrates the anti-ischemic stroke functions and mechanisms of Epimedium is provided. [file 3858314.f1.zip › supplementary file-revised.docx]

**Supplementary Materials**


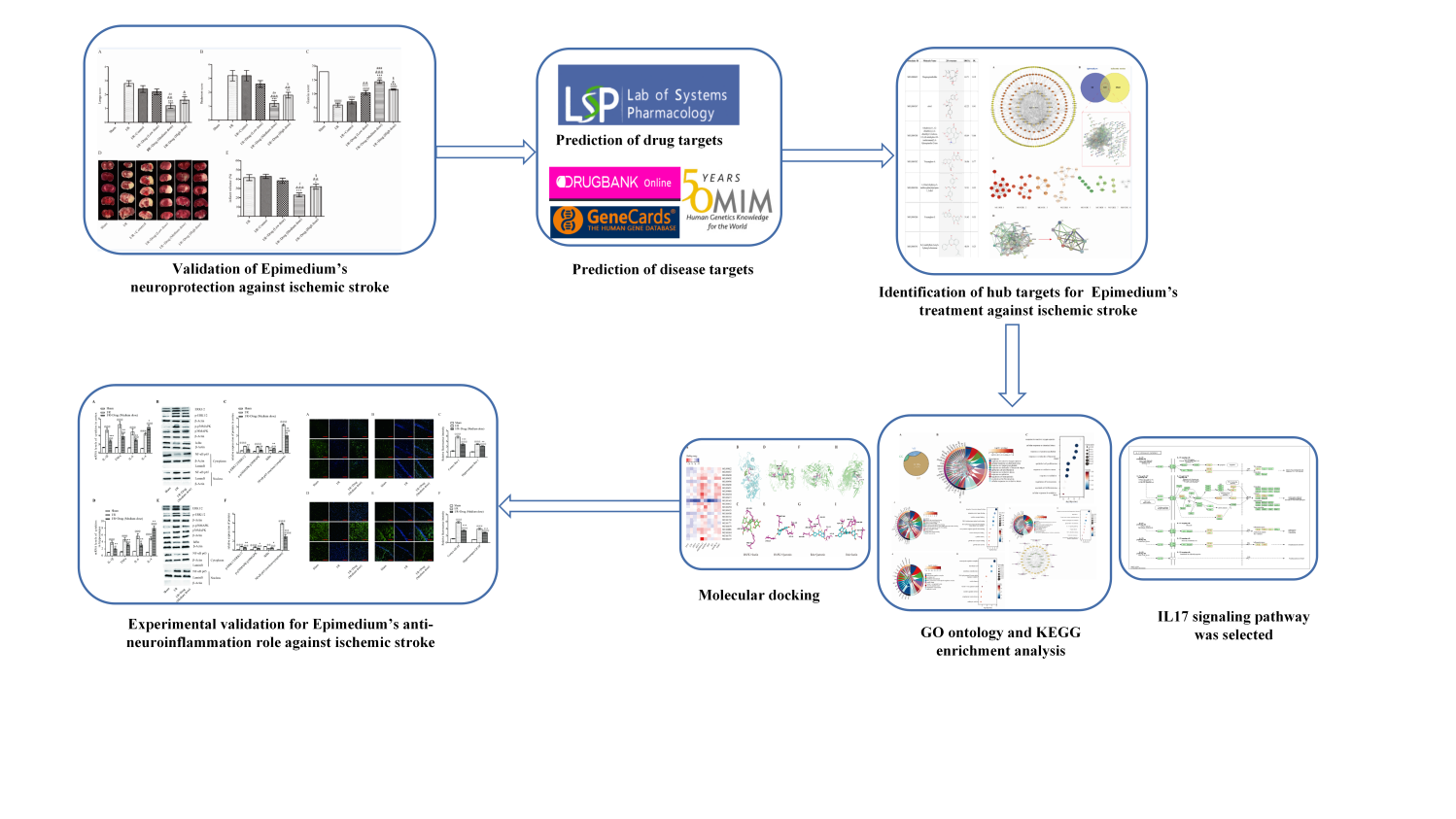
 Supplementary 1 The flowchart of this study.

Supplementary 2 PPI network of the 30 hub targets.
